# Effects of foot massage on relieving pain, anxiety and improving quality of life of patients undergone a cervical spine surgery

**DOI:** 10.1186/s12955-021-01667-2

**Published:** 2021-01-19

**Authors:** Nana Ren, Guangmin Yang, Xiaofeng Ren, Lekun Li

**Affiliations:** Department of Spinal Surgery, Liaocheng People’s Hospital, No. 67 Dongchang Xi Road, Liaocheng, 252000 Shandong China

**Keywords:** Cervical spine surgery, Foot massage, Pain and anxiety relief, Quality of life

## Abstract

**Background:**

Long-term recovery of patients undergone cervical spine surgery is of paramount importance to improve their quality of life. In this study we aimed to evaluate the effects of foot massage on relieving pain and anxiety of patients with anterior cervical discectomy and fusion (ACDF).

**Methods:**

Enrolled patients undergone ACDF and diagnosed with anxiety disorder at least six months before surgery were treated with 10-min foot massage on a daily basis for four weeks using sweet almond oil. Patients were assessed by neck pain visual analog pain scale (NP-VAS), neck disability index (NDI) and self-rating anxiety scale.

**Results:**

More significant relief in NP-VAS was observed in patients who received foot massage treatment. No significant difference in NDI reduction was seen in patients with or without the treatment. Intervention group demonstrated less anxiety during follow-up (*p* = 0.021) compared to the control group and more reduction compared to baseline (*p* = 0.046). In terms of quality of life, while both groups demonstrated improvement in pain relief (*p* = 0.015 for the intervention group and *p* = 0.037 for the control group), only the intervention group showed improved mental function (*p* = 0.031).

**Conclusion:**

This study found that foot massage was effective in alleviating pain and anxiety, while improving quality of life in patients undergone ACDF, indicating that this intervention should be considered in the clinical management of these patients.

## Introduction

Anterior cervical discectomy and fusion (ACDF) represents one of the most frequently conducted spinal procedures. ACDF is a widely-used procedure to treat several cervical spine pathologies, such as prolapsed intervertebral disc spondylosis, degenerative disc disease and trauma [1]. ACDF is in general associated with optimistic clinical outcomes and the majority of patients demonstrate a substantial reduction in neck pain; nevertheless, this procedure may lead to post-operative complications. For example, dysphagia is found to be present in 38.85% of patients at one month after ACDF [2]. This is concomitant with reports showing that post-operative mental health impairment, psychological distress and anxiety are common consequences of emotional factor following post-operative pain [3, 4]. It is also reported that as high as 47.3% of patients with dysphagia exhibit anxiety and depression [5]. Together, pain and anxiety worsen surgical outcomes and deteriorate quality of life in patients [6]. However, post-operative mental and psychological impairment caused by ACDF has not received enough attention, and strategies for management of pain and anxiety after ACDF surgery are in need for the benefit of these patients.

Massage therapy has increasingly been used as a technique to promote the mobilization of structures such as muscles and subcutaneous tissues, through the appliance of mechanical force to tissues [7]. Massage therapy induces venous return and lymph movement, decreases swelling, meanwhile mobilizes the skin, tendons and muscle fibers. Therefore, the effects of massage therapy include reduction of pain, stress and anxiety, as well as induction of muscle relaxation, which are shown to help improve quality of sleep and speed of recovery. Furthermore, massage therapy could enhance mobility, allowing patients to perform daily activities, which facilitates rehabilitation [8, 9].

Complementary intervention following surgery is increasingly considered effective and acceptable alternative management of pain. Massage therapy is a complementary intervention, which has been shown to relax patients and improve quality of life of patient undergone thoracic surgery or cardiac surgery [9, 10]. Accompanying this, patients showed improved clinical outcomes, reduced pain, less anxiety, fatigue, depression, anger and confusion.

Compared with massage on other body parts, foot message is a form of complementary intervention suitable for patients with ACDF as potential harm imposed by the message on the spine is avoided. To date, limited studies have been reported regarding the effects of foot massage in alleviating anxiety and pain in patients with ACDF. There is a lack of clear evidence on the effectiveness of foot massage, which precludes recommending this routine in the postoperative care of patients undergone ACDF. To clarify the benefit of foot massage in clinical practice, herein we aimed to conduct a randomized clinical trial to evaluate whether patients who received a four-week foot massage experienced less pain and anxiety and improved quality of life. Our results could provide valuable information to guide the clinical management of patients undergone ACDF.

## Patients and methods

The study was approved by the Ethics Committee of Liaocheng people’s Hospital. Consent forms were acquired from all patients. A total of 118 patients undergone ACDF during the period of 2016–2018 in Liaocheng People’s Hospital were assessed for eligibility. The inclusion criteria were as follows: (1) age over 18 years, (2) administration for over 1 day after surgery, (3) received an open reduction and internal fixation surgery. Since long-term diabetes is thought to have significant negative impact on spinal surgery [11] and cerebrovascular, neurological and psychiatric conditions are important factors to be assessed in our study, subjects with a history of diabetes for over 10 years or had a cerebrovascular, neurological, or psychiatric diseases were excluded. Among the 118 patients, 32 were excluded and 11 did not meet the inclusion criteria. 14 refused to participate and seven were not included for other reasons.

Randomization was performed using block randomization by a research scientist blind to the study. Equal numbers (n = 43 each group) of patients were assigned to either intervention group or control group. In the intervention group, massage treatment lasted four weeks. The treatment time we chose was mainly based on the time in hospital of the patients. In our hospital, the patients with anterior cervical discectomy and fusion are usually discharged one months after operation.

### Foot massage

On the second day after surgery, patients in the intervention group were guided to lay in a supine position and given a whole-body relaxation procedure, which lasted for five minutes. Then each foot was given 5–10 min of massage using sweet almond oil, which is the most commonly used lubricant in massage. During the massage, the subjects’ feet were elevated using a pillow. The massage procedure was performed on both sides of the foot (plantar and dorsal sides), such that the fingers were placed on the dorsal side of the foot and the thumbs on the plantar side. Then, the plantar region of the foot was pressed with one thumb, stroking upward using less pressure. This was done first on the heel and was later continued on the toes, with the plantar side of the foot held facing the therapist so that the fingers of the therapist were placed on the dorsal side of the toe. This procedure was repeated 5–10 times in a single massage session. An experienced therapist performed massage for all patients to ensure consistent therapy. Routine care was also given to both groups of patients. The patients did not receive massage on other parts of the body. The massage was conducted every other day from 4 weeks.

### Evaluation of pain, anxiety and quality of life

The questionnaires for evaluating pain, anxiety and quality of life were filled out by patients both before the intervention and after the 4-week intervention. Patients were guided to report neck pain assessed by neck pain visual analog scale (NP-VAS), in which patients were guided to indicate the pain intensity on a straight horizontal line of fixed length with the ends being defined as the extreme limits of pain intensities [12, 13]. The function of the neck was measured using Neck Disability Index (NDI), a questionnaire with 10 items regarding pain, personal care, lifting, reading, headaches, concentration, work, driving, sleeping and recreation [13].

Self-rating anxiety scale (SAS)[14] was also reported by the patients using a questionnaire consisted of 20 items, with a score of from 1 to 4 for each item. Their reported anxiety condition was categorized into: no anxiety (score < 50), mild anxiety (score = 50–59), moderate anxiety (score = 60–69) and severe anxiety (score > 69).

Assessment of quality of life was conducted using the QUALEFFO-41 subscales as the QUALEFFO‐41 subscales have been well validated to assess quality of life regarded to the orthopedic patients [15–17]. The QUALEFFO‐41 subscales consist of 41 questions to produce a total score with separate subscales for physical function, pain, general health perception, social function and mental function [18]. The total score and each subscale give a maximum score of 100, with higher scores representing increased severity.

### Statistical analysis

Parametric data were presented as mean ± standard deviation (SD) and compared via the Student’s t-test or Wilcoxon signed rank test as appropriate. Non-parametric data were presented as mean and *p* values were derived from Chi-square test or Fisher’s exact test. Effect size was calculated using Phi, which can be computed by finding the square root of the chi-squared statistic divided by the sample size. Clinical relevant differences were set at effect size ≥ 0.4.

## Results

**Fig. 1:**
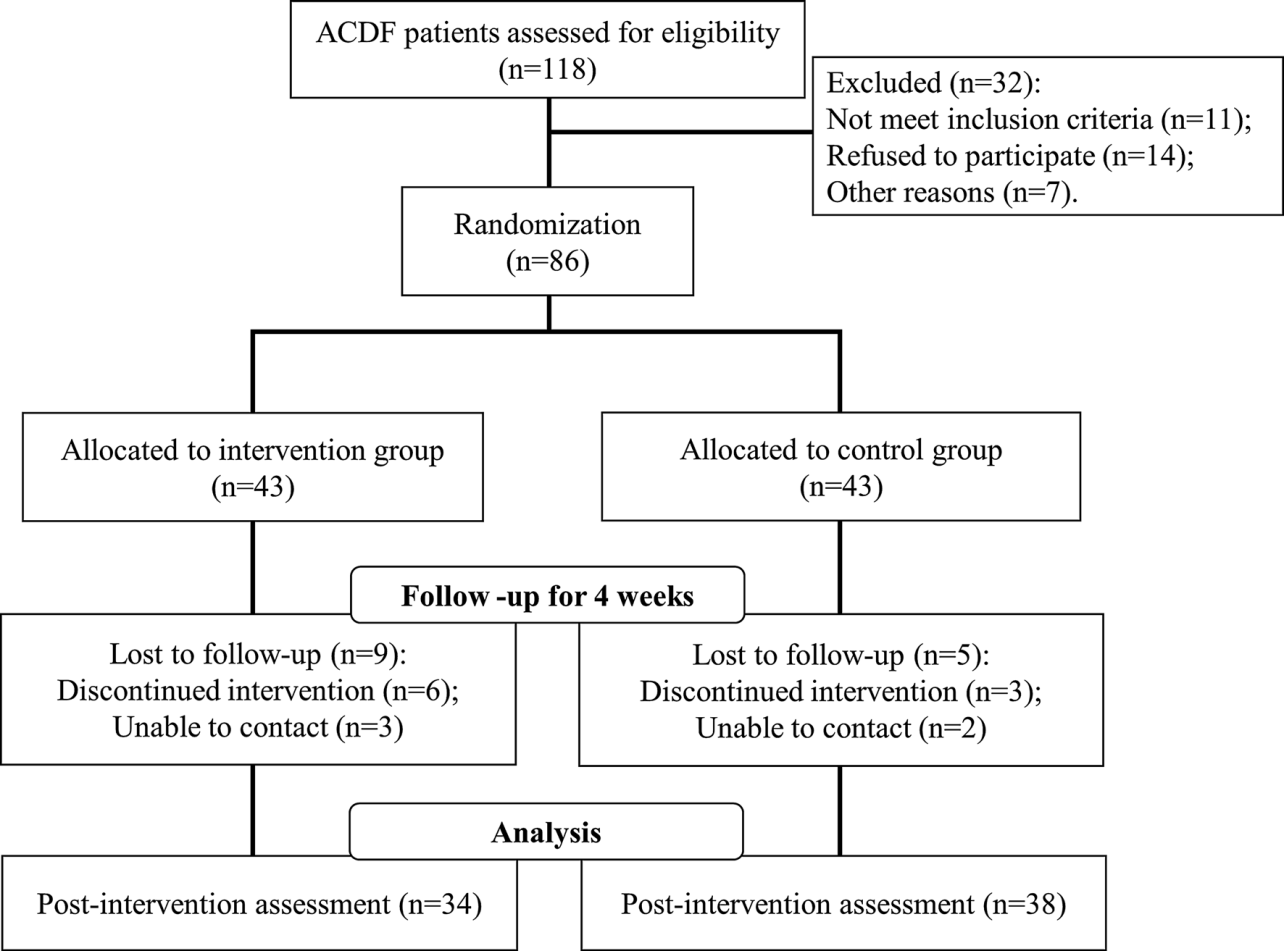
Research framework of this study

A total of 118 patients were enrolled in this study and 86 patients were assigned randomly to the intervention group (n = 43) and the control group (n = 43) (Fig. 1). As shown in Table 1, the patients in the two groups did not show significant differences in baseline characteristics including gender, age, body mass index (BMI), percentage of patients who smoke, with hypertension, diabetes, coronary artery disease, or hyperlipidemia, as well as education. The average age was 54.2 ± 9.4 years in the intervention group and 52.8 ± 12.4 years in the control group, and the majority of the patients were female.

**Table 1:** Demographic characteristics of the patients analyzed

Variable	Study group		*p*
	Intervention group (n = 34)	Control group (n = 38)	
Age (years)	54.2 ± 9.4	52.8 ± 12.4	0.246
Male gender, n (%)	15 (44.1%)	14 (36.8%)	0.632
BMI	28.9 ± 6.9	30.2 ± 7.5	0.195
Preoperative duration of pain, months	16.2 ± 14.3	13.8 ± 16.1	0.352
Smoker, n (%)	11 (32.4%)	16 (42.1%)	0.468
Hypertension, n (%)	13 (38.2%)	10 (26.3%)	0.319
Diabetes, n (%)	8 (23.5%)	13 (34.2%)	0.437
CAD, n (%)	5 (14.7%)	4 (10.5%)	0.726
HLD, n (%)	7 (20.6%)	10 (26.3%)	0.592
Education			
Junior high school and below	16 (47.1%)	14 (36.9%)	0.314
Senior high school or polytechnic school	12 (35.3%)	20 (52.6%)	
College and above	6 (17.6%)	4 (10.5%)	

Values were expressed as n (percentage, %) or mean ± SD. *p* values for each group were derived from either unpaired t test or Mann–Whitney test as appropriate. Chi-square test or Fisher’s exact test was used for assessing distribution of observations or phenomena between different groups

BMI: body mass index; CAD, coronary artery disease; HLD, hyperlipidemia

### Foot massage alleviates neck pain and neck disability

**Fig. 2:**
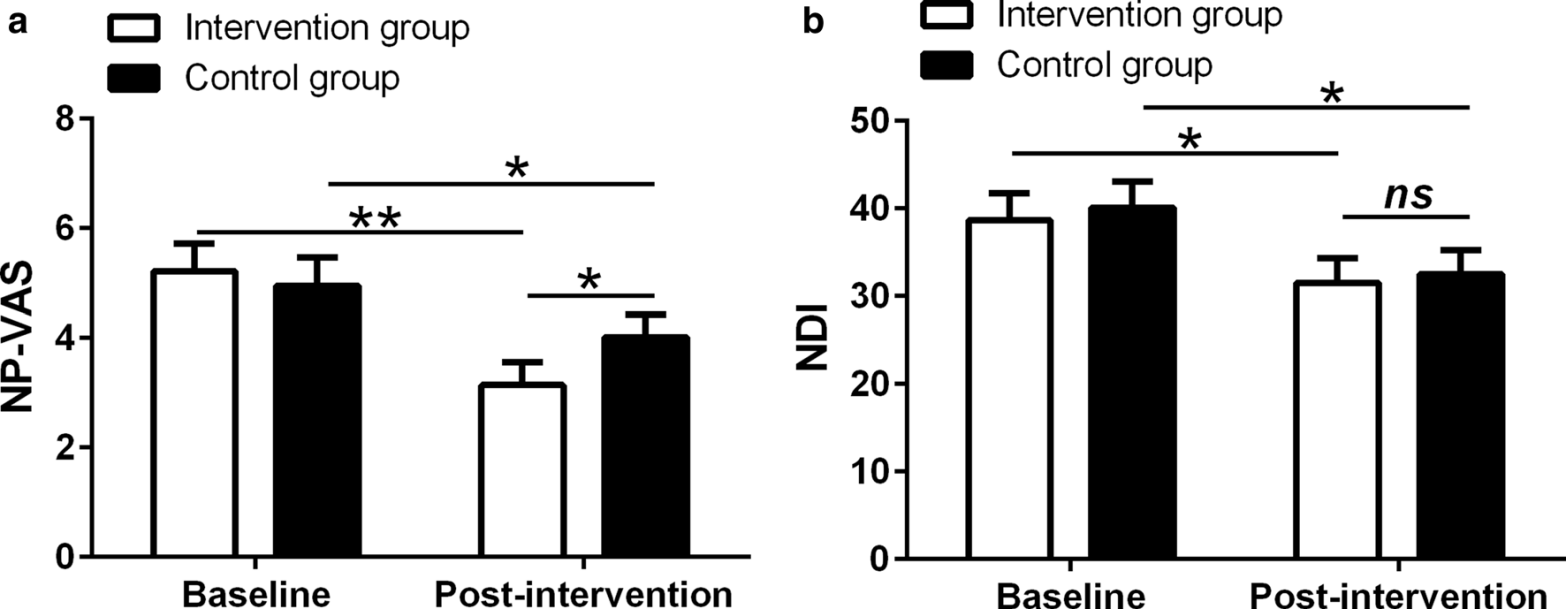
Effects of 4-week foot massage on neck pain visual analog pain scale (NP-VAS) (**a**) and neck disability index (NDI) (**b**) in patients having undergone the surgery of anterior cervical discectomy and fusion (ACDF)

We first evaluated the effects of foot massage in alleviating neck pain and neck disability after ACDF. As shown in Fig. 2a, b, both groups demonstrated lower NP-VAS score (*p* < 0.01 for the intervention group and *p* < 0.05 for the control group) (Fig. 2a) and NDI score (Fig. 2b) (*p* < 0.05 for both groups). However, the intervention group showed lower NP-VAS in the follow-up period compared to the control group (*p* < 0.05).

### Foot massage reduces anxiety after surgery

Since we enrolled patients with short-term anxiety because of injury and surgery, it is important to evaluate whether foot massage could reduce anxiety among the patients. Patients were asked to give a SAS score, which was based on a scoring system consisted of 20 items. As reported in Table 2, we found that only the intervention group showed a significant reduction in anxiety compared to baseline (*p* = 0.046), and the post-intervention score was remarkably lower in the intervention group compared to the control group (*p* = 0.021).

**Table 2:** Comparison of SAS before and after intervention

Self-rating anxiety scale (SAS)	Study group			
	Intervention group (n = 34)		Control group (n = 38)	
	Baseline	Post-intervention	Baseline	Post-intervention
Not anxiety (< 50)	12 (35.3%)	23 (64.7%)	14 (36.8%)	12 (31.6%)
Mild anxiety (50–59)	11 (32.4%)	7 (23.5%)	10 (26.3%)	14 (36.8%)
Moderate anxiety (60–69)	6 (17.6%)	3 (5.9%)	8 (21.1%)	10 (26.3%)
Severe anxiety (> 69)	5 (14.7%)	1 (5.9%)	6 (15.8%)	2 (5.3%)
*p* value (at the same time compared to control group)	0.949	0.021		
*p* value (in the same group compared to baseline)	0.046		0.385	

Values were expressed as n (percentage, %). *p* values were derived from Chi-square test or Fisher’s exact test was used for assessing distribution of observations or phenomena between different groups

### Foot massage improves quality of life

In terms of quality of life, we assessed patients in terms of general physical function, pain, social function, mental function and general health perception using the QUALEFFO-41 scores (Table 3). We found that, while both the intervention and control groups showed reduction in general pain, the post-intervention pain score of the intervention group was markedly lower than the control group (*p* = 0.041). In addition, only the intervention group showed a significant reduction in mental function compared to baseline. The total scores of the two groups were not significantly different.

**Table 3:** Assessment of quality of life before and after the intervention

QUALEFFO-41 (0–100)		Study group		*p* value
		Intervention group (n = 34)	Control group (n = 38)	
Pain	Baseline	78.8 ± 14.1	81.4 ± 16.4	0.168
	Post-intervention	58.3 ± 13.6	67.7 ± 11.6	0.041
	*p* value	0.015	0.037	
Physical function	Baseline	61.4 ± 15.9	59.7 ± 17.4	0.354
	Post-intervention	52.3 ± 16.7	49.7 ± 18.1	0.176
	*p* value	0.176	0.089	
Social function	Baseline	56.7 ± 14.8	57.6 ± 15.1	0.531
	Post-intervention	50.1 ± 17.9	52.8 ± 16.7	0.605
	*p* value	0.115	0.131	
General health perception	Baseline	68.7 ± 20.1	72.5 ± 18.9	0.251
	Post-intervention	55.9 ± 17.8	59.7 ± 21.6	0.226
	*p* value	0.068	0.059	
Mental function	Baseline	58.2 ± 13.1	60.5 ± 11.2	0.398
	Post-intervention	44.9 ± 11.4	50.9 ± 14.9	0.067
	*p* value	0.031	0.085	
Total score	Baseline	58.7 ± 15.8	59.1 ± 17.3	0.634
	Post-intervention	51.3 ± 16.9	53.9 ± 14.4	0.139
	*p* value	0.116	0.153	

Values were expressed as mean ± SD. *p* values derived from paired t test or Wilcoxon signed rank test as appropriate between baseline versus post-intervention. *p* values derived from unpaired t test or Mann–Whitney test as appropriate between intervention group and Control group

## Discussions

In the search for a more effective postoperative management of ACDF, psychological factors in addition to pain, such as anxiety should be considered. Postoperative anxiety has been associated with longer hospital stay, higher re-admission rates and dissatisfaction with the received care [4]. However, to our best knowledge, limited studies have been conducted to evaluate postoperative massage on the pain and anxiety management of ACDF.

Here we showed that patient who received foot massage demonstrated markedly alleviated anxiety. Emerging studies have suggested that relaxation, which can be induced in a variety of ways, affects pain. Foot massage is gaining popularity as an approach for pain management. In the present study, we showed that foot massage was associated with decreased neck pain and disability in patients undergone ACDF. Consistent with our study, Eghbali et al. demonstrated that after arthroscopic knee surgery, massage on the healthy foot, hands, and upper shoulder relieved pain [19]. Another report also showed that massage was effective in alleviating acute pain in post-thoracic surgery [10]. Massage has a long history of being used to treat a variety of illnesses. However, empirical and theoretical support in utilizing massage as a complementary intervention to alleviate anxiety, which usually concurs with pain, is thus far limited. In chemotherapy of cancer, complementary relaxation therapy has been shown to reduce anxiety and facilitate the recovery of patients, as well as improve quality of life [20]. A number of clinical studies have also confirmed that postoperative massage is powerful in improving the quality of life in patients undergone cardiac surgery [21–23].

In contrast to a study by Adogwa et al. on preoperative treatment of anxiety in ACDF management, which showed that preoperative treatment of anxiety did not improve NDI, thereby exerting no functional benefit [24], we showed that postoperative foot massage was associated with significantly improved NDI. Our study is the first one to demonstrate significant reduction in both pain and anxiety following ACDF using foot massage as a complementary therapy. Other possible strategies for pain and anxiety management include the use of anti-depressants [25] or mood-altering drug [26] such as duloxetine [27], which however is inferior in the low-cost and safety of massage.

Our evaluation was based on questionnaires to provide numeric values in the assessment of pain, anxiety, disability and general health state, which nonetheless lacks clinically significant meaning. Thus, in the study by Parker et al. on ACDF, the minimal clinical important difference (MCID) has been introduced to measure the critical threshold of achieving treatment effectiveness [28]. Here we did not associate the clinical outcomes with foot massage intervention, to make definitive conclusions on how foot massage intervention would affect the clinical outcome, the use of MCID may be needed. Further, randomization was not stratified by gender. BMI and the number of smokers in the control group were also higher than those in the intervention group, which, albeit insignificant, may affect the conclusions of the study. Another limitation is that a relatively short follow-up period (four weeks) was used and long-term effects of foot massage in postoperative management of ACDF should be investigated. Further studies are also needed to evaluate whether foot massage would benefit other complications of ACDF, such as sleep disorders.

## Conclusion

Here we have demonstrated that postoperative foot massage for four weeks is associated with effectively reduced neck pain and neck disability, relieved anxiety and improved quality of life in patients. These findings suggest that foot massage is worth being used as a complementary care for patients undergone ACDF.

## Data Availability

The study data is available upon request.

## Abbreviations

ACDF: Anterior cervical discectomy and fusion
NP-VAS: Neck pain visual analog pain scale
NDI: Neck disability index
SAS: Self-rating anxiety scale
